# Nonsense-Mediated mRNA Decay Immunity Can Help Identify Human Polycistronic Transcripts

**DOI:** 10.1371/journal.pone.0091535

**Published:** 2014-03-12

**Authors:** Guy Shahaf, Dorit Shweiki

**Affiliations:** Bioinformatics Program, School of Computer Science, The Academic College of Tel Aviv-Yaffo, Tel Aviv, Israel; CNRS UMR7622 & University Paris 6 Pierre-et-Marie-Curie, France

## Abstract

Eukaryotic polycistronic transcription units are rare and only a few examples are known, mostly being the outcome of serendipitous discovery. We claim that nonsense-mediated mRNA decay (NMD) immune structure is a common characteristic of polycistronic transcripts, and that this immunity is an emergent property derived from all functional CDSs. The human RefSeq transcriptome was computationally screened for transcripts capable of eliciting NMD, and which contain an additional ORF(s) potentially capable of rescuing the transcript from NMD. Transcripts were further analyzed implementing domain-based strategies in order to estimate the potential of the candidate ORF to encode a functional protein. Consequently, we predict the existence of forty nine novel polycistronic transcripts.

Experimental verification was carried out utilizing two different types of analyses. First, five Gene Expression Omnibus (GEO) datasets from published NMD-inhibition studies were used, aiming to explore whether a given mRNA is indeed insensitive to NMD. All known bicistronic transcripts and eleven out of the twelve predicted genes that were analyzed, displayed NMD insensitivity using various NMD inhibitors. For three genes, a mixed expression pattern was observed presenting both NMD sensitivity and insensitivity in different cell types. Second, we used published global translation initiation sequencing data from HEK293 cells to verify the existence of translation initiation sites in our predicted polycistronic genes. In five of our genes, the predicted rescuing uORFs are indeed identified as translation initiation sites, and in two additional genes, one of two predicted rescuing uORF is verified. These results validate our computational analysis and reinforce the possibility that NMD-immune architecture is a parameter by which polycistronic genes can be identified. Moreover, we present evidence for NMD-mediated regulation controlling the production of one or more proteins encoded in the polycistronic transcript.

## Introduction

The vast majority of eukaryotic genes are considered monocistronic with a single transcription unit encoding for a single protein (alternatively-spliced variants included). Polycistronic transcription units (no trans-splicing involved; i.e., "eukaryotic operon") are rare in eukaryotes and specifically in mammals, and therefore little is known on how they differ from the monocistronic ones.

Genomically organized polycistronic units are known in several organisms (e.g., nematodes, Arabidopsis thaliana) yet those are trans-spliced and each monocistronic unit is translated independently [1]. Further, episodic occurrences of eukaryotic bicistronic transcripts, which do not undergo trans-splicing are documented (including STNA-STNB in Drosophila; GK-GPR in tomato and mammalian GDF-1-LASS1, SNRPN-SNURF, MTPN-LUZP6 and MFRP- C1QTNF5) [1], [2], [3], [4], [5].

Newly synthesized mRNAs are subjected to a pioneer round of translation in which premature termination codon (PTC) containing transcripts are identified and degraded in various degrees of efficiency via the Nonsense-mediated mRNA decay (NMD) mechanism [6], [7]. In mammals, NMD onset is primarily associated with the identification of un-removed exon-junction protein complexes (EJCs) in PTC-containing transcripts [8]. During the pioneer round event, previously deposited splicing-dependent EJCs, positioned ∼20–24 nucleotides upstream to the exon-exon junction, are detached and removed. It was demonstrated that translating ribosomes are responsible for the removal of the EJCs positioned within the coding region, during the pioneer round of translation [9], [10], [11]. Un-removed EJCs in prematurely translation-terminated transcripts trigger NMD degradation. By and large, PTCs elicit NMD if positioned more than 55 nucleotides upstream to the terminal exon-exon junction, known as the “55 nucleotide rule”. Stop codons positioned downstream to this site (in the penultimate or the terminal exon) fail to elicit NMD and are considered NMD immune [7], [12].

Seven polypeptides constitute the mammalian NMD core mechanism: up-frameshift protein 1 (UPF1), UPF2, UPF3 (comprised isoforms UPF3 and UPF3X) SMG1, SMG5, SMG6 and SMG7. UPF1 is the most conserved, essential protein, with RNA-dependent ATPase and 5′-3′ helicase activities [13], [14]. UPF1 was shown to directly interact with both cap-binding-protein CBP80 and translation termination factors eRF1 and/or eRF3, thus likely linking NMD and translation termination activities [15], [16]. In the event of premature termination, UPF1 and SMG1 interact with EJC-associated UPF2 and UPF3X. Consequent to UPF1/SMG1- EJC interaction, SMG1-mediated UPF1 phosphorylation occurs, triggering translational repression and NMD induced degradation [17], [18]. Until recently the common belief was that NMD is restricted to the pioneer round of translation and only to mRNAs which are associated with cap-binding-protein CBP80-CBP20 complex. Following the removal of the EJCs and the CBP80-CBP20 complex and its replacement by eIF4E, the transcript therefore becomes NMD immune, free to undergo multiple translation cycles [14], [19], [20], [21]. Recently, however, several lines of evidences indicated that NMD may also occur on eIF4E-bound transcripts, which are already being translated [22], [23].

Turning a polycistronic transcript into NMD-immune following the pioneer round of translation requires the removal of all EJCs subsequent to the translation of the functional CDS. In this manuscript we raise the hypothesis that human (and most likely mammalian) functional polycistronic transcripts share a distinctive NMD-immune architecture, which is an emergent property of all functional CDSs. Further, we argue that the definition of potentially NMD-eliciting transcripts (failing to fulfill the "55 nucleotide rule") ought to include the 5' UTR of the molecule, as occurs in many upstream open reading frame (uORF) containing transcripts. In non-polycistronic transcripts. uORFs, which are found in almost 50% of human genes, are mainly characterized by their negative regulatory effect [24], [25], [26], [27]. In other words, the upstream CDS in polycistronic transcripts and the regulatory uORF differ by their NMD-induction potential.

A computational-based approach was utilized to survey monocistronic and polycistronic transcript architecture and to predict the existence of novel polycistronic transcripts in the human transcriptome. We screened the human RefSeq dataset for potentially NMD-eliciting transcripts, according to the classic definition and our modified one. Further, we aimed to isolate those transcripts containing ORFs capable of "rescuing" the mRNA from its NMD-eliciting destiny, i.e., overlapping the exon junctions or positioned in their proximity (as detailed in the Methods section). We then applied domain-based strategies (see below) to predict the potential of the candidate ORF to encode a functional protein. Polycistronic (mainly bicistronic) transcript prediction is presented and discussed.

## Results

### Known human polycistronic transcript architecture

**Figure 1:**
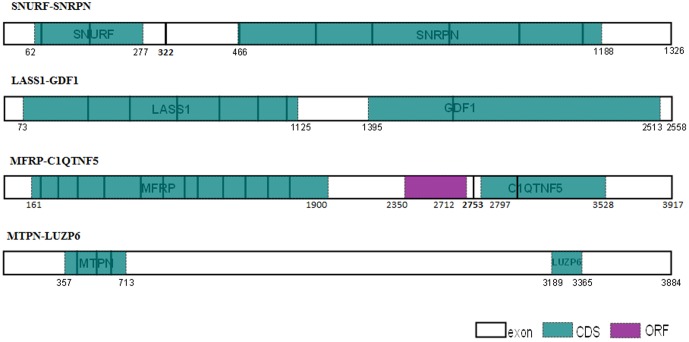
Known human polycistronic transcripts architecture. Exon junctions highlighted in bold, uncovered exon junction coordinates are indicated in bold; annotated CDS in turquoise; ORF in purple; CDS, ORF and transcript coordinates are indicated.

Our main hypothesis was that polycistronic transcripts share a distinctive NMD-immune architecture, leading to the production of stable mRNA, maximally available for translation. We thus assessed the architecture of the known human polycistronic and bicistronic transcripts (Figure 1). In two bicistronic genes (LASS1-GDF1, and SNURF-SNRPN), NMD immunity is hypothesized to be contributed by both CDSs (LASS1-GDF1, all exon junctions are covered by the two CDSs; SNURF CDS ends 45 nucleotides upstream to the exon junction). In the MFRP-C1QTNF5 gene, three ORFs are responsible for NMD-immune architecture, yet only two of them are documented to encode the known proteins (the first encodes MFRP and the last - C1QTNF5). The MFRP-C1QTNF gene was found to be strongly expressed in human medulla oblongata [3]. Thus, we speculated that, either the middle ORF is also translated, contributing to the removal of the EJCs, or that EJC removal results from an upstream ribosome-dependent spatial effect, occurring during translation reinitiation of the C1QTNF5 CDS (with its AUG positioned only 44 nucleotides downstream to the exon junction; see discussion section). Finally, the MTPN stop codon in the MTPN-LUZP6 transcripts is positioned in the terminal exon solely responsible for transcript NMD-immunity, while LUZP6 is encoded by a cryptic ORF positioned in the 3′ UTR region, which uses IRES and a non-AUG translation initiation codon [5]. Hence, in the four known human bicistronic examples, NMD-immune architecture is demonstrated. Yet, given the very few documented genes available, more evidence is necessary.

### Novel polycistronic transcript prediction in the 3' UTR of penultimate or upstream NMD-eliciting transcripts

Based on the architecture of the known bicistronic transcripts, we devised a strategy for the identification of novel polycistronic genes. Polycistronic transcript search was limited to potentially NMD-eliciting transcripts with an annotated stop codon positioned in the penultimate or upstream exon. Transcripts in which the annotated stop codon is positioned in the terminal exon (similar to the MTPN-LUZP6 gene) were excluded from this study due to the following reasons: (i) the vast majority of the known mammalian bicistronic genes share an NMD-immune architecture contributed by all functional CDSs; (ii) no other criteria were indicative enough: our preliminary results show that ORF coding potential alone is insufficient to distinguish functional ORFs from non-functional ones (data not shown). Furthermore, comparative genomics *per se* seem to be inadequate based on the lack of evolutionary conservation in the known bicistronic genes.

In all, 30035 Refseq records were analyzed for potentially failing to fulfill the “55 nucleotides rule” and eliciting NMD, as detailed in the Methods section. Of these, 113 transcripts contained an annotated stop codon positioned 55 nucleotides or more upstream to the terminal exon-exon junction. Those were further analyzed for the existence of ORFs which are potentially capable of turning the transcript from NMD-eliciting into NMD-immune. Ninety three potential rescuing ORFs were identified in 68 transcripts.

The existence of a rescuing ORF overlapping an exon junction is far from sufficient in order to identify a polycistronic transcript. We therefore assessed potential functional ORFs based on the following criteria: (i) Existence of a translation initiation sequence. Two potential elements - Kozak-like sequence and internal ribosome entry sites (IRESs) in the 5' end can be considered. We avoided relying on IRES identification as a search criterion because its presence in cellular mRNAs is still debated [28], [29]. Over 85 reported cellular IRES-containing transcripts share long 5' UTRs, multiple uAUGs and a similar GC content, yet a considerable amount of genes fit this profile with no evidences for IRES existence [29], [30]. Furthermore, IRESs are characterized structurally, with no known consensus sequence and therefore *in silico* identification is problematic, and most studies focus on empirical data validation not on novel IRES prediction [30], [31], [32]. Indeed, when screening the known bicistronic transcripts for IRES sequences, utilizing UTRScan and IRSS, no IRES elements were identified (data not shown) [33], [34]. Still, all polycistronic candidates reported in this manuscript were computationally analyzed for IRES elements with no positive results. (ii) No significant similarity between the candidate ORF sequence and the annotated CDS (or CDSs of alternatively spliced isoforms of the same gene; lower than 50% similarity). A high degree of sequence similarity was assumed to indicate gene rearrangement rather than the existence of a functional ORF. (iii) The potential ORF encoded protein shares a significant similarity to other proteins in the protein database or contains functional domains according to InterProScan analysis (or both – see Methods). In addition, candidate polycistronic transcripts were screened for transcript architecture conservation in other organisms, utilizing BLAST analysis to GenBank databases.

Out of the 93 potential rescuing ORFs, 53 (39 transcripts) were discarded due to high homology between the rescuing ORF and the annotated CDS. The remaining ORFs were further analyzed according to the criteria elaborated above. Eight candidate bicistronic transcripts (6 genes) were identified, out of which two were discarded because the predicted protein was identified to contain only a signal peptide sequence, with no other known protein domains (See Methods section). From the remaining six transcripts, three novel (2 genes) and three known bicistronic transcripts (SNRPN, MFRP and LASS1; GI's: 29540557, 223633880 and 110349723, respectively) were identified (Table 1, only novel candidates are presented).

**Table 1:** Novel bicistronic transcript candidates followed 3′ UTR analysis of penultimate or upstream NMD-eliciting transcripts.

Gene Symbol	Gene Name	Transcript GI	Predicted functional ORF position	Kozak Sequence	InterProScan	BlastP
C20orf203	Chromosome 20 open reading frame 203	292658848	1876..2109	CTTACTATGT	signal peptide 1–19; PTHR12138, family-not-named domain 11–49	No
NAT15	N-acetyltransferase 15 (GCN5-related, putative)	134254454	1165..1716	CAGAGCATGC	signal peptide 1–23;	95% identity with hypothetical protein LOC100609520 [Pan troglodytes] 183 a.a.; 86% identity with hypothetical protein LOC100443079 [Pongo abelii] 223 a.a.
		134254439	1115..1666			

### Annotated ATG exon position: implications for polycistronic transcripts prediction

Limiting the search for functional ORFs to the 3' UTR of the mRNA seems arbitrary. One CDS may indeed be more dominant over the other in terms of its expression level, yet it is not necessarily the first in the polycistronic transcript (e.g., SNURF-SNRPN). Similar to the strategy undertaken in the former stage, we needed to distinguish transcripts which contain a regulatory uORF from polycistronic ones in which the upstream CDS is still unknown. The upstream CDSs in polycistronic transcripts and regulatory uORFs differ first and foremost by their NMD-induction potential. Thus we performed a preliminary analysis aiming to identify potentially NMD-eliciting transcripts based on mRNA 5' screening. We analyzed the distribution of the annotated ATG exon position in human RefSeq transcripts and evaluated how many of them are potentially NMD-eliciting (unless a rescuing ORF will be revealed). NMD degradation induction relies on EJCs that remain after the pioneer round of translation. Since no known sequence-based parameters are available to indicate whether translation re-initiation will occur in sequential ORFs, our approach is applicable only for those cases in which the uORF/CDS and the annotated ATG are positioned in different exons and therefore at least one remaining EJC potentially exists. Transcripts for which the first exon contains the 5′ UTR and the annotated ATG, as well as potentially encoding ORF, were not included in our study as they require experimental evaluation of re-initiation and NMD-eliciting potential. We found that only 59% of the annotated ATGs are positioned in the first exon of the transcript and the rest are positioned in the second or downstream exons (Table 2).

**Table 2:** Annotated ATG exon position in human RefSeq transcripts.

Exon no.	1	2	3	4	5	6	7	8	9	10	11	12	13	14
No. of Transcripts	17715	8908	2369	687	217	77	35	15	6	1	1	1	1	2
% of Total	59.0	29.7	7.9	2.3	0.72	0.26	0.12	0.050	0.020	0.003	0.003	0.003	0.003	0.007

Transcripts in which the annotated ATG is positioned in the second or downstream exons were analyzed for 5′ UTR ORF existence (12320 records; 41% of the Refseq transcriptome). Of these, 6118 transcripts (20.3% of total Refseq transcripts) contain no ORF in their 5′ UTR, i.e., the ribosomal 43S pre-initiation complex is assumed to scan the mRNA until the annotated ATG is reached (detaching pre-deposited EJCs on its way) [19], [20], [21]. These transcripts were therefore not considered as candidates for 5′ UTR-related NMD-induction. Another 4130 transcripts (13.8% of total Refseq transcripts), contain one or more ORFs in their 5′ UTR, yet are expected to display NMD-immunity due to their architecture which theoretically ensures removal of all EJCs according to the “55-nucleotide rule”, implemented on the 5′ UTR. Finally, 2063 records (6.9% of total Refseq transcripts), although they contain an ORF in their 5′ UTR, are predicted to have an NMD-eliciting architecture, since not all EJCs are expected to be removed after the pioneer round of translation, even if re-initiation occurs at the annotated ATG.

At this point we concluded the following: (i) a considerable portion of RefSeq transcripts contain two or more ORFs, are in NMD-immune architecture, and therefore have the potential to function as polycistronic mRNAs; (ii) NMD-eliciting potential in the human transcriptome is likely higher then so far evaluated, due to 5′ UTR-related NMD-eliciting architecture (2063 records; 6.9% of total Refseq transcripts).

### 5′ UTR-based novel polycistronic transcript prediction

After dividing the transcriptome into groups according to the annotated ATG position and the existence of rescuing uORFs, we turned to predict the 5' UTR-related novel polycistronic transcript potential. A total of 4130 transcripts (13.8% of Refseq transcriptome) constitute the dataset from which we aimed to differentiate transcripts with regulatory uORFs from those with functional upstream CDSs.

Two working assumptions guided this stage: (i) the first ATG identified by the 43S pre-initiation complex can be positioned in the second and downstream exon, and all EJCs deposited upstream to it are removed. Therefore no full exon-junctions coverage is required, and instead we screened for exon-junction coverage between the end of the first ORF identified and the annotated ATG. (ii) potential ORFs were analyzed only if the ORF was larger than 99 nucleotides. This cutoff value was set based on the size range of known polycistronic encoded proteins (59 to 580 amino acids, LUZP6 and MFRP, respectively) and the relatively small size of human uORFs (average length 51.5 nucleotides) [35]. ORF encoding potential and Kozak sequence recognition were carried out as described above and in Methods. Out of the 4130 candidate transcripts screened, 335 were identified to contain ORFs larger than 99 nucleotides with no significant similarity between the candidate ORF sequence and the annotated CDS. Of these, 81 transcripts: (i) contain a Kozak-like sequence in proximity to the candidate AUG and (ii) the potential ORF-encoded protein shares a significant similarity to other proteins in the protein database and/or contains functional domains according to InterProScan analysis. In 29 of these 81 transcripts, InterProScan analysis identified only a signal peptide sequence and/or transmembrane regions, and they were therefore discarded (See Methods section). The remaining 52 transcripts are considered polycistronic candidates, among which three are known transcripts (SNURF-SNRPN, LUZP6 and GDF1; GIs: 29540556, 190886450 and 110349791, respectively). An additional three undergo an unusual transcription pattern: leptin receptor (LEPR, GI: 310923183), which is reported to share the same promoter and the first two exons with the leptin receptor overlapping transcript (LEPROT) gene [36]; The IGF 2 read-through product (GI: 183603938); And the GPR75- ASB3 gene (G protein-coupled receptor 75-ankyrin repeat and SOCS box containing 3; GI: 188528701) read-through product [37] (Table 3, a detailed description in Table S1).

**Table 3:** Novel human polycistronic transcript candidates followed 5' UTR analysis.

GeneID	Gene Symbol	Gene Name
80823	BHLHB9	basic helix-loop-helix domain containing, class B, 9
6046	BRD2	bromodomain containing 2
84798	C19orf48	chromosome 19 open reading frame 48
9139	CBFA2T2	core-binding factor, runt domain, alpha subunit 2; translocated to, 2
966	CD59	CD59 molecule, complement regulatory protein
9425	CDYL	chromodomain protein, Y-like
56616	DIABLO	diablo, IAP-binding mitochondrial protein
405754	ERVFRD-1	endogenous retrovirus group FRD, member 1
57579	FAM135A	family with sequence similarity 135, member A
391059	FRRS1	ferric-chelate reductase 1
**2657**	**GDF1**	**growth differentiation factor 1**
81491	GPR63	G protein-coupled receptor 63
**10936**	**GPR75**	**G protein-coupled receptor 75**
3146	HMGB1	high mobility group box 1
**3481**	**IGF2**	**insulin-like growth factor 2 (somatomedin A)**
3781	KCNN2	potassium intermediate/small conductance calcium-activated channel, subfamily N, member 2
**3953**	**LEPR**	**leptin receptor**
401052	LOC401052	hypothetical LOC401052
**767558**	**LUZP6**	**leucine zipper protein 6**
8195	MKKS	McKusick-Kaufman syndrome
318	NUDT2	nudix (nucleoside diphosphate linked moiety X)-type motif 2
5569	PKIA	protein kinase (cAMP-dependent, catalytic) inhibitor alpha
11272	PRR4	proline rich 4 (lacrimal)
80758	PRR7	proline rich 7
5724	PTAFR	platelet-activating factor receptor
494115	RBMXL1	RNA binding motif protein, X-linked-like 1
5265	SERPINA1	serpin peptidase inhibitor, clade A (alpha-1 antiproteinase, antitrypsin), member 1
6579	SLCO1A2	solute carrier organic anion transporter family, member 1A2
**6638**	**SNRPN**	**small nuclear ribonucleoprotein polypeptide N**
441273	SPDYE2	speedy homolog E2 (Xenopus laevis)
1E+08	SPDYE2L	WBSCR19-like protein 3
442578	STAG3L3	stromal antigen 3-like 3
51807	TUBA8	tubulin, alpha 8
347736	TXNDC6	thioredoxin domain containing 6
9724	UTP14C	UTP14, U3 small nucleolar ribonucleoprotein, homolog C (yeast)
9189	ZBED1	zinc finger, BED-type containing 1
9189	ZBED1	zinc finger, BED-type containing 1
51351	ZNF117	zinc finger protein 117
8187	ZNF239	zinc finger protein 239
339324	ZNF260	zinc finger protein 260
353274	ZNF445	zinc finger protein 445
55769	ZNF83	zinc finger protein 83
162962	ZNF836	zinc finger protein 836
284371	ZNF841	zinc finger protein 841

Novel polycistronic transcript candidates are presented (alphabetically sorted by gene symbol). Documented genes highlighted in bold.

### Novel polycistronic transcript validation

We hypothesized that human polycistronic mRNAs share a unique configuration, in which functional CDSs are mutually organizes in an NMD-immune structure. This architecture was demonstrated in four known bicistronic genes (LASS1-GDF1, SNURF-SNRPN, MFRP-C1QTNF5 and MTPN-LUZP6), and was further used to predict the existence of 49 novel polycistronic transcripts. In order to validate our predictions, we screened the literature and databases for known cases of NMD inhibition and transcription initiation site detection.

#### NMD insensitivity of polycistronic transcripts

mRNA expression datasets from Gene Identification by NMD inhibition (GINI) experiments, in which mRNA levels are compared in the presence and absence of NMD- inhibitors (emetine, caffeine and NMD-specific siRNA inhibitors) were utilized to identify polycistronic transcript NMD sensitivity. Five datasets representing a variety of cell types were downloaded from the Gene Expression Omnibus database (GEO) and analyzed. Tables 4 and 5 summarize the NMD sensitivity status of the known bicistronic (Table 4) and polycistronic predicted genes (Table 5; detailed information in Tables S2A and S2B) found in the different experiments. Overall, the known bicistronic genes display considerable, stable expression in the different cell types analyzed (Table 4, Table S2A). Fourteen of the predicted genes fulfilled our primary criterion, i.e. genes which all their documented transcripts seem polycistronic (see Methods section). Out of these, twelve are represented in the various datasets that were used for validation (C20orf203, ERVFRD-1, FRRS1, HMGB1, LOC401052, LOC442578, MGC119295, STAG3L3, TXNDC6, UTP14C, ZNF117 and ZNF841), mostly displaying NMD insensitivity (Table 5, Table S2B). ZNF841 exhibits NMD sensitivity in the sole experiment that monitored this gene, in mononuclear leukocytes taken from both healthy and prostate cancer patients (GSE24204). Three more genes display partial NMD sensitivity: The UTP14C gene displays a mixed expression pattern in different cell types, with NMD sensitivity in one experiment and insensitivity in three others. The FRRS1 gene exhibits NMD insensitivity in one experiment (GSE16170) using two treatments (Ago2 siRNA - targets CBP80/20-bound mRNAs and thus considered a regulator of NMD and UPF1 and Ago2 siRNA), and NMD sensitivity in another experiment, in mononuclear leukocytes of prostate cancer patients but not of healthy patients (GSE24204). Finally, the HMGB1 gene displays a mixed expression pattern, indicating NMD sensitivity in one out of 4 experiments. Yet in the same experiment, one HMGB1 probe produces an NMD sensitivity pattern while the other probe indicates NMD insensitivity (GSE29788). The probe which is responsible for the NMD sensitivity pattern identifies also an additional antisense transcript (AF83771), for which we have no CDS annotation, and thus may be an artifact. It may be argued that NMD inhibition is not be fully achieved in GINI experiments and thus lead to somewhat distorted results, yet several observations oppose this notion, including: different methods of NMD inhibition were undertaken in the various experiments analyzed; the mixed results of NMD sensitivity and insensitivity, which was limited to a narrow number of our genes and not for the entire array; and significant, high levels of expression displayed for only some of our genes. All these characteristics do not fit a general NMD-inhibition failure pattern.

**Table 4:** NMD sensitivity status of human bicistronic genes in published NMD-inhibition experiments.

GEO Dataset/Cell type	Citation	NMD -Inhibition method	Gene symbol	ProbeID	Type of transcripts identified	NMD Sensitivity
GSE1703 Hela Cells	Mendell, JT. et al, Nat Genet. 36, 1073 - 1078 (2004); PMID:15448691	RENT1-siRNA	GDF1-LASS1	887_at	bicistronic transcripts (NM_0212673; NM_001492)	NMD insensitive
				888_s_at	monicistronic variant (NM_198207)	NMD insensitive
			SNRPN-SNURF	34842_at	both monicistronic and bicistronic variants	NMD insensitive
GSE16170 Hela Cells	Choe, J. et al EMBO Rep 11(5): 380-386 (2010); PMID: 20395958	Ago2 siRNA; UPF1 and Ago2 siRNA.	SNRPN-SNURF	ILMN_1656537	both monicistronic and bicistronic variants	NMD insensitive
			MTPN-LUZP6	ILMN_2180682	bicistronic transcript (NM_145808)	NMD insensitive
GSE20491 Clear cell renal cell carcinoma	Duns, G. et al, Cancer Res 70(11):4287–4291 (2010). PMID:20501857	Emetine or caffeine inhibition	SNRPN-SNURF	ILMN_1660000	bicistronic transcript (NM_005678)	NMD insensitive
			MTPN-LUZP6	ILMN_218068, ILMN_1791478	bicistronic transcript (NM_145808)	NMD insensitive
GSE24204 Prostate cancer	Mattila, H., University of Tampere. Finland (unpublished).	Emetine inhibition	GDF1-LASS1	25143	two bicistronic transcripts (NM_0212673; NM_001492)	NMD insensitive
			MFRP-C1QTNF5	37231, 20996	bicistronic transcripts (NM_031433; NM_015645)	NMD insensitive
			MTPN-LUZP6	4388, 23064, 41236	bicistronic transcript (NM_145808)	NMD insensitive
GSE29788 Head and neck cell lines	Sharma. S., et al, Mol Cancer Ther. 10(9):1751–1759, (2011). PMID: 21764905	Emetine inhibition	SNRPN-SNURF	201522_x_at, 206042_x_at	both monicistronic and bicistronic variants	NMD insensitive

**Table 5:** NMD sensitivity status of human polycistronic predicted genes in published NMD-inhibition experiments.

GEO Dataset/Cell type	Citation	NMD -Inhibition method	Gene symbol	ProbeID	Type of transcripts identified	NMD Sensitivity
GSE1703 Hela Cells	Mendell, JT. et al, Nat Genet. 36, 1073–1078 (2004); PMID:15448691	RENT1-siRNA	ZNF117	36783_f_at	NM_015852.	NMD insensitive
			UTP14C	39405_at	UTP14C (chr13) and UTP14 (chrX) genes.	NMD insensitive
GSE16170 Hela Cells	Choe, J. et al EMBO Rep 11(5): 380–386 (2010); PMID: 20395958	Ago2 siRNA; UPF1 and Ago2 siRNA.	HMGB1	ILMN_2231242	NM_002128	NMD insensitive Both with Ago2 siRNA and UPF1 + Ago2 siRNAs.
			UTP14C	ILMN_1686645	NM_021645	
			FRRS1	ILMN_2214734	NM_001013660	
			LOC401052	ILMN_1791423	NM_001008737	
			MGC119295	ILMN_2144654	NM_001031618	
			LOC442578	ILMN_1791375	NM_001013739	
GSE20491 Clear cell renal cell carcinoma	Duns, G. et al, Cancer Res 70(11):4287–4291 (2010). PMID:20501857	Emetine or caffeine inhibition	HMGB1	ILMN_223124; ILMN_1791466	NM_002128	NMD insensitive. (both emetine and caffeine; in 10 cell lines)
			UTP14C	ILMN_1686645	NM_021645	NMD insensitive (both emetine and caffeine; in 10 cell lines)
GSE24204 Prostate cancer	Mattila, H., University of Tampere. Finland (unpublished). Healthy and cancerous cells	Emetine inhibition	C20orf203	27463	AK091025	NMD insensitive
			HMGB1	27795, 2170, 7063, 8395	NM_002128	NMD insensitive
			UTP14C	32662	NM_021645	***NMD sensitive***
			ZNF841	39976	NM_001136499	***NMD sensitive***
			TXNDC6	7699, 11753, 4719	NM_178130	NMD insensitive
			FRRS1	31823	NM_001013660	NMD insensitive in healthy cells; ***NMD sensitive in cancer cells***
			LOC401052	13485	NM_001008737	NMD insensitive
			ERVFRD-1	14886	NM_207582	NMD insensitive
			STAG3L3	3563	NM_001013739	NMD insensitive
GSE29788 Head and neck cell lines	Sharma. S., et al, Mol Cancer Ther. 10(9):1751–1759,(2011) PMID: 21764905	Emetine inhibition	HMGB1	200679_x_at; 200680_x_at	NM_002128	NMD insensitive
				214938_x_at	NM_002128 and AF283771 - anti-sense transcript	***NMD sensitive***
			UTP14C	203614_at	NM_021645	NMD insensitive
			ZNF117	207117_at; 207605_x_at	NM_015852	NMD insensitive

#### Translation initiation site (TIS) detection in predicted polycistronic transcripts

NMD is not 100% efficient [6], [7]. Consequently, NMD insensitivity validation *per se* is not sufficient, as some transcripts may show detectable levels of seemingly stable mRNA even though NMD degradation does occur. A more direct verification is therefore desired, reinforcing our validation results. We therefore crosschecked our predicted transcript list with a dataset of experimentally verified TISs, produced by utilizing global translation initiation sequencing (GTI-seq) in the transcriptome of human embryonic cell line (HEK293) [38]. Out of the 49 polycistronic transcripts predicted in this study, 9 transcripts (5 genes) are listed in the Lee *et al*. TIS dataset, in the exact position and with the same ORF size as predicted in our study (Table 6). Additionally, two more transcripts, in which two uORFs are expected to rescue the transcript from NMD, are partially listed in this dataset with only one TIS verified.

**Table 6:** Human polycistronic transcripts found in Lee *et al* TIS dataset: Novel polycistronic transcripts candidates that were found in Lee *et al* TIS dataset with exact match both in ORF start position and length; missing rescuing ORF in brackets.

GeneID	Gene Symbol	RefSeq Accession (GI)	Predicted ORF	ORF size	Line No. in Table S1	Line No. in Lee et al Table S1
3953	LEPR	NM_001003680 (310923183)	74..184	111	22	9113
		NM_002303 (310923184)			23	9114
		NM_001003679 (310923185)			24	9112
8195	MKKS	NM_018848 (25914751)	261..452	192	28	19993
9189	ZBED1	NM_004729 (57165426)	43..165	123	45	22240
		NM_001171136 (283806700)	43..168	126	46	22242
80823	BHLHB9	NM_030639 (216547631)	101..211	111	4	19752
		NM_001142528 (216547671)	101..226	126	5	19742
494115	RBMXL1	NM_001162536 (242247050)	378..548	171	35	10363
84798	C19orf48	NM_199249 (40548381)	[139..243] 337..378	42	7	21003
339324	ZNF260	NM_001166036 (260436927)	201..299 [390..485]	99	49	8477

## Discussion

The function of NMD in quality control surveillance and as a gene expression regulatory apparatus is well documented. Diverse events contribute to PTC occurrence, with a key role for alternative splicing, nonsense mutations and SNP-related events [39], [40], [41], [42]. The regulatory aspect of NMD was demonstrated in several physiological settings including the regulation of selenoprotein mRNAs, splicing-factor gene expression, physiologically-related classes of transcripts in Hela cells and others [14], [43]. Additionally, a role for an exon-truncated class of alternative polyadenylation as an NMD-rescue regulatory mechanism was previously suggested by us [44].

The NMD mechanism is not 100% efficient. Up to 25% of the PTC-containing transcripts escape NMD degradation [6], [7]. An efficient re-initiation site within a short distance of a nonsense mutation and in the same exon, was demonstrated to elicit NMD rescue and immunity of the transcripts [45], [46]. At times, escape was shown to be associated with the regulatory mechanism responsible for the introduction of the PTC (e.g. ApoB48 and thrombopoietin translation control) [47], [48]. Overall, 5.8% protein isoforms of the SWISSPROT database are derived from PTC containing transcripts, indicating its regulatory effect [49]. Hence, the existence of a PTC in a given transcript does not necessarily indicate the transcript's destiny, but may rather hint on regulatory stratification.

The novelty of our work is in suggesting a yet undescribed connection between NMD and polycistronic gene architecture. In this study we hypothesized that human polycistronic mRNAs (and most likely mammalian ones in general) share a unique configuration, in which functional CDSs are mutually organized in an NMD-immune architecture. Indeed, we detected NMD-immune transcript architecture in the known human bicistronic genes. Further, we analyzed the human Refseq transcriptome, predicting the existence of 49 novel polycistronic transcripts. Bioinformatics-based analysis, indicating the existence of known protein domains in the predicted functional-ORFs encoded proteins or their similarity to known proteins, supports our assumption regarding the encoded proteins' potential to be produced and active. mRNA expression datasets from GINI experiments were utilized for experimental verification, aiming to explore whether the allegedly polycistronic transcripts are insensitive to NMD. All the known bicistronic transcripts and an additional eleven genes from our predicted gene list displayed NMD insensitivity for various NMD inhibitors, including emetine, caffeine and UPF1 and Ago2 siRNAs (other genes in our list were not sampled in those GINI experiments). The gene ZNF481, which was studied in a single experiment, displayed an NMD sensitive profile. An additional three of the eleven genes displayed a mixed expression pattern of both NMD sensitivity and insensitivity in different cell types, possibly manifesting time-dependent and process-dependent translation re-initiation regulation. Furthermore, some of the genes displayed significantly high levels of expression which do not fit the expression pattern of inefficient NMD. Hence, these results reinforce our view that an NMD-immune architecture is likely to play a role in polycistronic transcript expression regulation. Finally, we succeeded to find 9 predicted polycistronic transcripts (5 genes) in a translation initiation sites dataset, with the exact position and ORF size as predicted in our study (and 2 additional partial matches, for a total of 7 genes) [38]. No overlap between these gene lists of validated polycistronic transcripts was found. Yet, it is expected that different cell types will exhibit different patterns of polycistronic gene regulation, thus, one does not exclude the other. Altogether, 16 novel polycistronic genes are experimentally validated.

The rationale for novel polycistronic transcript prediction was based on distinguishing functional CDSs from regulatory ORFs by their potential to elicit NMD and to encode a functional protein. Many studies addressed the question of parameters affecting translation re-initiation following a uORF, including uORF size, length and lack of secondary structure of the intercistronic spacing sequences and the usage of conserved uATG [19], [25], [26], [50], [51], [52], [53]. Yet no sequence-based information is clear enough to pinpoint whether translation re-initiation will occur. Thus, and although many additional parameters may play a role, and other polycistronic models are likely to exist, no sequence-based models, other than PTC occurrence seem usable. Our work therefore provides one possible scenario arguing for the existence of cellular polycistronic transcripts with an NMD-immune architecture, permitting both mRNA stability and a regulatory mean to control the expression of all or some of the functional CDSs.

It was shown that following translation of a uORF and the release of the 60S subunit, the 40S subunit may remain on the mRNA and resume scanning for as far as 600 nucleotides, without re-initiating translation [54]. Consequently, theoretically, EJCs may be removed from downstream exon junctions in the absence of near-by translation re-initiation. Undoubtedly, this observation is of importance for searching for an NMD-immune architecture, yet the lack of additional knowledge on the conditions affecting 40S scanning and EJC removal prevents us from implementing this knowledge in our study.

Almost half of the human genes have uORFs in their 5′ UTRs, capable of reducing protein expression by 30 to 80% [24], [53], [55], [56], [57]; and though these findings are well documented and acknowledged, they are not assimilated into the vast majority of studies screening and evaluating the NMD fraction of the transcriptome in different contexts. In this study we challenged the classical definition of the "55 nucleotide rule", arguing that it should be "stretched" to the 5' UTR of the transcripts. Namely, we claim that the search for polycistronic-related functional ORFs should take place both in the 3' and 5' UTRs, upstream and downstream to the annotated CDS. Subsequently, we estimate the fraction size of NMD-eliciting transcripts in human Refseq transcriptome to be approximately 7.3%, significantly larger in comparison to its size (0.4%) when analyzing only the 3' UTR.

Moreover, we further argue that while evaluating the potential for NMD, both sides of the exon-exon junction (upstream and downstream) ought to be equally considered. EJCs deposited 20–24 nucleotides upstream to the exon junction are being pushed away and removed by the ribosome. The ribosome's spatial dimensions dictate a downstream EJC displacement even if the stop codon is positioned 50 to 55 nucleotides upstream to the exon junction. Based on this fact, we argue that in the event of translation re-initiation, EJC removal is likely to occur even if the ORF starts in close proximity downstream to the exon-exon junction, further changing NMD fraction size as estimated computationally. Indeed, yeast ribosome footprint experiments indicated the protection of 28 nucleotides upstream and downstream to the ATG (−12 to +15 nucleotides) [58]. Taking into account EJC size and the re-initiation-related sequence being scanned by the ribosome, a region larger than 12 nucleotides is likely to be covered. Possibly, this claim is demonstrated in the known human bicistronic gene MFRP-C1QTNF5, whose transcript contains 3 ORFs contributing to the NMD-immune architecture of the transcript. Yet the gene is recognized by its first and last encoded proteins. There is no evidence for the translation of the protein encoded in the central ORF. We think that it is possible that the last ORF is responsible for removal of the EJCs during translation, due to ribosomal spatial hindrance, even though its ATG is positioned 44 nucleotides downstream to the exon junction. Although the MFRP-C1QTNF5 gene potentially supports our view, the distance range downstream to the exon junction ought to be examined and established experimentally before being implemented in computational studies.

We assume that many functional-ORF encoded proteins are expressed in low levels or to a limited period of time, thus their detection is challenging. Moreover, we believe that seemingly NMD-eliciting, polycistronic transcripts are underrepresented in the mammalian genome annotation, partially due to the tendency to suppress NMD candidate mRNAs from nucleic databases (e.g., RefSeq database policy). NMD-eliciting transcripts seem to include remarkable regulatory features, waiting to be further studied. We therefore hope that our hypothesis will be further verified by experimentalists.

## Methods

### Human transcriptome dataset

35157 Human Refseq mRNAs records were downloaded from the RefSeq database (NCBI, Release dated 18/12/2010; http://www.ncbi.nlm.nih.gov/RefSeq/) [59]. 1286 records were discarded due to unavailable information on their exon positions or joint CDS in their annotation. Records with annotated stop codon coordinates positioned after the last exon were discarded for 3' analysis (14 records).

### NMD-candidate identification

The remaining 33871 records were analyzed for their potential to elicit NMD. Stop codon and exon-intron partitioning of the mRNA molecule was retrieved based on the Refseq annotation.

The annotated stop codon was identified as PTC if the 3′ most nucleotide of the stop codon is positioned more than 55 nucleotides upstream of the terminal exon–exon junction. Transcripts in which the stop codon is positioned in the terminal exon or in the 55 nucleotides preceding the terminal exon junction were considered as NMD-immune. 113 records were identified to potentially elicit NMD.

### ORF identification in 3' UTRs

"Rescuing ORFs", capable of turning NMD-eliciting transcripts into NMD-immune one, were searched screening the mRNA sequence in the three reading-frames (0, +1, +2) from 5' to 3' direction. In order for an ORF to be defined as a rescuing one, all exon-exon junctions, downstream to the annotated stop codon, are to be covered according to the "55 nucleotide rule". Namely, the ORF should cover the entire sequence length within the range of 55 nucleotides upstream to the first exon-exon junction-downstream to the annotated stop codon, and up to at least 55 nucleotides upstream to the terminal junction (or further downstream). For NMD-eliciting transcripts harboring an annotated stop codon in the penultimate exon in a distance >55 nucleotides, a minimal rescuing ORF should start and end in the penultimate exon in a distance smaller than 55 nucleotides upstream to the terminal exon. If more than one ORF was required in order to turn NMD-eliciting transcript into NMD-immune, they were considered as one unit.

Out of 113 NMD-eliciting transcripts, 68 NMD-immune transcripts contained one or more rescuing ORFs (a total of 93 ORFs). At this stage, all rescuing ORFs were considered, including ORFs which overlap one another in different or identical reading-frames. No overlap was allowed between the annotated CDS and rescuing ORFs.

### ORF identification in 5' UTRs

Functional uORFs were searched for in the 5′ UTRs of RefSeq records for which the annotated CDS start codon is in the second or later exon. An ORF screen was carried out in the three reading-frames (0, +1, +2) from 5' to 3'. Records which followed our hypothesized polycistronic architecture, namely, exon-junctions coverage between the end of the first ORF identified and the annotated ATG and potential ORFs larger than 99 nucleotides, were further analyzed. Out of 4130 transcripts containing ORFs in their 5' UTR with an NMD-immune architecture, 335 transcripts were identified to contain ORFs larger than 99 nucleotides and with no significant similarity between the candidate ORF sequence and the annotated CDS, and were further evaluated for polycistronic and bicistronic candidacy.

### Kozak sequence element

Initiation of translation by eukaryotic ribosomes is optimal at the ACCATGG consensus sequence [60]. Yet a purine in position −3 (relative to the A nucleotide in the ATG) followed by ATG at positions +1-+3 is sufficient for efficient translation initiation, thus this minimal sequence was identified as Kozak positive in this study.

### BLAST analysis

BLASTN analysis was carried in order to rule out candidate ORF encoded proteins with significant similarity to the annotated protein of the transcript. ORF sequences were analyzed against the human RefSeq mRNA dataset utilizing BLASTN standalone application with default parameters [61]. No significant similarity between the candidate ORF sequence and the annotated CDS (or CDSs of alternatively spliced isoforms of the same gene) was allowed. A high degree of sequence similarity was hypothesized to indicate a 3′ UTR ORF, which is the result of a gene rearrangement event rather than the existence of a functional ORF. Parsing of the BLASTN results was based on a threshold of e-value greater than 1.00E-06 and 50% or more coverage between the CDS from transcripts with the same gene ID and the candidate ORF.

### Functional ORF encoded protein characterization utilizing BLASTP

Candidate functional ORFs were predicted based on whether the potential ORF encoded protein shares a significant similarity to other proteins in the protein database. Candidate ORFs were translated to potential protein sequences and were analyzed for protein similarity utilizing BLASTP (standalone version) with default parameters against the non-redundant protein sequences (nr) database [61]. A positive ratio (number of positive hits/ORF length) > 0.5 or align length (alignment length/ORF length) > 0.8 was used to identify significant hits.

### Functional ORF encoded protein characterization utilizing InterProScan

Further, candidate functional ORFs were predicted based on whether the potential ORF contains functional domains according to InterProScan analysis. The ORF-encoded proteins were screened for known protein domains utilizing InerProScan Web Services (http://www.ebi.ac.uk/Tools/InterProScan/) [62].

The signal peptide domain and the transmembrane domain were considered insufficient to predict a functional protein. Although the subcellular localization of proteins is widely studied with many tools, its prediction is not always accurate. Further, non-classical secretion pathways exist, assisting in the secretion of signal-peptide free proteins, contributing to the uncertainty of predicting subcellular localization [63]. Thus, identifying polycistronic transcript existence based on a signal peptide or transmembrane domain existence seems less reliable, and additional indications were required.

### Polycistronic transcripts NMD-immunity validation

Five GINI experiments datasets (GSE1703, GSE16170, GSE20491, GSE24204 and GSE29788) in which mRNA levels are compared in the presence and absence of NMD- inhibitors of different sorts (i.e., the chemicals emetine and caffeine and NMD-specific siRNA inhibition; see Tables 4 and 5 for experiment treatment details), were downloaded from the GEO database [64]. Experiments were selected based on data enabling reanalysis, and at least partial overlap between the known and predicted bicistronic genes and probes represented in the array. mRNA expression results were used only if the probe/s available in the array identified the only gene of question and not additional gene family members. Further, since the vast majority of human genes undergo alternative splicing, we obviously preferred relying on probes which distinguish between monocistronic and polycistronic transcripts of the same gene, when such probes were available. For the predicted genes, we limited ourselves to genes which all their documented transcripts (according to RefSeq annotation), are polycistronic according to our prediction (one or more). If threshold expression levels are defined in the dataset annotation (published manuscripts included), those were taken under consideration, and otherwise all available results were included. In order to assess whether gene expression levels significantly differ between NMD-inhibition treatment and control cells, we implemented heteroscedastic two tailed T-Test analysis. For those experiments which specifically set a cut-off value defining the threshold of NMD sensitivity, we used the later (as indicated in Tables 4 and 5 and in Tables S2A and S2B).

## Supporting Information

Table S1 **Novel polycistronic transcript candidates followed 5' UTR analysis.**
(XLSX)

Table S2 **Table S2A - Known human bicistronic genes in published GINI experiments.** Table S2B - Predicted polycistronic genes in published GINI experiments.
(PDF)
