# Supplementary material for: Nonsense-Mediated mRNA Decay Immunity Can Help Identify Human Polycistronic Transcripts
Source: PLoS One. 2014 Mar 12;9(3):e91535. doi: 10.1371/journal.pone.0091535 (PMC3951408; doi:10.1371/journal.pone.0091535)
Supplement: Table S2 — A - Known human bicistronic genes in published GINI experiments. Table S2B - Predicted polycistronic genes in published GINI experiments. (PDF) [file pone.0091535.s002.pdf]

Table S2A: Known human bicistronic genes in published GINI experiments

<table>
<tr><th>GEO dataset</th><th>Citation / NMD inhibition method / microarray</th><th>Gene symbol</th><th>ProbeID [I]</th><th>Results (average expression value)</th><th>T-Test Analysis</th></tr>
<tr><td rowspan="3">GSE1703</td><td rowspan="3">Mendell, JT. Et al, Nat Genet. 36, 1073 - 1078 (2004)
PMID:15448691

Hela cells; RENT1-siRNA treated

Affymetrix Human Genome U95 Version 2 Array</td><td rowspan="2">GDF1-LASS1</td><td>887_at
Identifies the two bicistronic transcripts of this gene (NM_0212673; NM_001492)</td><td>control Hela cells - 330.5;
RENT1-siRNA cells - 365.5</td><td>0.58</td></tr>
<tr><td>888_s_at
Identifies monicistronic variant of this gene (NM_198207)</td><td>control Hela cells - 173.85;
RENT1-siRNA treated cells - 180.7</td><td>0.76</td></tr>
<tr><td>SNRPN-SNURF</td><td>34842_at
Identifies both monicistronic and bicistronic variants of this gene[II]</td><td>control Hela cells - 580.75;
RENT1-siRNA treated cells - 527.4</td><td>0.051</td></tr>
<tr><td rowspan="2">GSE16170</td><td rowspan="2">Choe, J. et al EMBO Rep 11(5): 380-386 (2010)
PMID: 20395958

HeLa, Cos-7 and Huh-7 cells; Ago2 siRNA and UPF1 and Ago2 siRNA treated.

Illumina HumanRef-8 v3.0 expression beadchip</td><td>SNRPN-SNURF</td><td>ILMN_1656537
Identifies both monicistronic and bicistronic variants of this gene[II]</td><td>control cells – 10.46 [III]
Ago2 siRNA treated cells - 10.40
UPF1 and Ago2 siRNA treated cells - 10.44</td><td>control – Ago2 0.83
Control-UPF1+Ago2 0.89</td></tr>
<tr><td>MTPN-LUZP6</td><td>ILMN_2180682
Identifies the bicistronic transcript NM_145808[IV].</td><td>control cells – 12.56 [III]
Ago2 siRNA treated cells - 12.75
UPF1 and Ago2 siRNA treated cells - 12.69</td><td>control – Ago2 0.18
Control-UPF1+Ago2 0.345</td></tr>
<tr><td>GSE20491</td><td>Duns, G. et al, Cancer Res 70(11):4287-4291 (2010) [V].
PMID:20501857

10 clear cell renal cell</td><td>SNRPN-SNURF</td><td>ILMN_1660000 probe identifies the NM_005678 bicistronic variant solely and therefore displayed [III]. [6 probes in this array identify monocistronic and bicistronic variants of this gene]</td><td><table>
<tr><th>Cell line</th><th>Control Average</th><th>Caffeine Average</th><th>Emetine Average</th></tr>
<tr><td>RCC1</td><td>7.4</td><td>7.7</td><td>7.0</td></tr>
<tr><td>RCC4</td><td>7.6</td><td>7.1</td><td>7.2</td></tr>
<tr><td>RCC5</td><td>8.0</td><td>7.5</td><td>7.6</td></tr>
<tr><td>RCC-AB</td><td>6.7</td><td>-</td><td>6.7</td></tr>
</table></td><td><table>
<tr><th>control-caffeine</th><th>control-emetine</th></tr>
<tr><td>0.12</td><td>0.02</td></tr>
<tr><td>0.25</td><td>0.28</td></tr>
<tr><td>0.18</td><td>0.23</td></tr>
<tr><td>-</td><td>0.63</td></tr>
</table></td></tr>
</table>

	carcinoma cell lines; emetine or caffeine treated.<br><br>Illumina HumanHT-12 V3.0 expression beadchip			RCC-ER	8.6	8.2	8.3	0.13	0.26	
				RCC-FG2	6.9	7.0	6.9	0.10	0.97	
				RCC-HS	9.0	9.0	8.4	0.66	0.04 [VI]	
				RCC-JF	8.8	8.7	7.7	0.88	0.00 [VI]	
				RCC-JW	7.9	8.0	7.3	0.60	0.13	
				RCC-MF	6.9	6.6	7.2	0.04	0.09	
		MTPN-LUZP6	ILMN_2180682 and ILMN_1791478 identify the bicistronic transcript NM_145808[IV].	Cell line	Control Average	Caffeine Average	Emetine Average	control-caffeine	control-emetine	
				RCC1	11.1	10.8	9.5	0.74	0.17	
				RCC4	10.4	9.9	9.4	0.55	0.24	
				RCC5	10.5	10.2	9.4	0.78	0.33	
				RCC-AB	11.2	-	10.1	-	0.18	
				RCC-ER	10.4	10.1	9.1	0.75	0.19	
				RCC-FG2	10.4	10.9	9.6	0.84	0.60	
				RCC-HS	11.0	10.5	10.1	0.61	0.39	
				RCC-JF	10.7	10.4	9.4	0.66	0.09	
				RCC-JW	11.1	10.7	9.9	0.71	0.28	
				RCC-MF	10.1	9.1	8.3	0.49	0.20	
GSE24204	Mattila, H., University of Tampere. Finland (unpublished)<br><br>peripheral mononuclear leukocytes cell lines from healthy and prostate cancer patients; Emetine treated cells; normalized log10 ratio emetine treated/control cells<br><br>Agilent-012391 Whole Human Genome Oligo	GDF1-LASS1	25143 - Identifies the two bicistronic transcripts of this gene (NM_0212673; NM_001492)	Healthy average expression ratio: -0.14<br>Cancer average expression ration: -0.19						In this study, a considered gene was declared differentially expressed if the calculated log10 ratio value was greater than zero. All our genes display values smaller than zero indicating on a down-regulation effect after exposure to emetine.
		MFRP-C1QTNF5	37231 - identifies both bicistronic transcripts of this gene (NM_031433 and NM_015645)	Healthy average expression ratio: -0.16<br>Cancer average expression ration: -0.19						
			20996 - identifies both bicistronic transcripts of this gene (NM_031433 and NM_015645)	Healthy average expression ratio: -0.18<br>Cancer average expression ration: -0.25						
		MTPN-LUZP6	4388 - identifies the bicistronic transcript NM_145808	Healthy average expression ratio: -0.33<br>Cancer average expression ration: -0.34						
			23064 - identifies the bicistronic transcript NM_145808	Healthy average expression ratio: -0.30<br>Cancer average expression ration: -0.36						

	Microarray G4112A		41236 - identifies the bicistronic transcript NM_145808	Healthy average expression ratio: -0.32<br>Cancer average expression ration: -0.35	
GSE29788	Sharma. S., et al, Mol Cancer Ther. 10(9):1751-1759,(2011)<br>PMID: 21764905<br><br>Primary human head and neck cancer cell line (SCC12) and matched normal controls; Emetine treated cells; probes which are marked as absent in all cells were not considered.<br><br>NEI <1.7 (nonsense enrichment<br>Index) indicate on NMD-immune transcript.<br><br>Affymetrix Human Genome U133A Array	SNRPN-SNURF	201522_x_at and 206042_x_at - Identifies both monicistronic and bicistronic variants of this gene.	201522_x_at - NEI=0.68<br>206042_x_at - NEI=0.66	

Table S2B: Predicted polycistronic genes in published GINI experiments

<table>
<tr><th>GEO dataset</th><th>Citation / NMD inhibition method / microarray</th><th>Gene symbol</th><th>ProbeID [I]</th><th>Results (average expression value)</th><th>T-Test Analysis</th></tr>
<tr><td rowspan="2">GSE1703</td><td rowspan="2">Mendell, JT. Et al, Nat Genet. 36, 1073 - 1078 (2004)
PMID:15448691

Hela cells; RENT1-siRNA treated

Affymetrix Human Genome U95 Version 2 Array [I]</td><td>ZNF117</td><td>36783_f_at
Identifies NM_015852.</td><td>control Hela cells – 37.2;
RENT1-siRNA cells – 22.75;</td><td>0.02</td></tr>
<tr><td>UTP14C</td><td>39405_at this probe identifies both UTP14C (chr13) and UTP14 (chrX) genes.</td><td>control Hela cells – 49.1;
RENT1-siRNA treated cells – 58.05</td><td>0.57</td></tr>
<tr><td>GSE16170</td><td>Choe, J. et al EMBO Rep 11(5): 380-386 (2010)
PMID: 20395958

HeLa, Cos-7 and Huh-7 cells; Ago2 siRNA and UPF1 and Ago2 siRNA treated.

Illumina HumanRef-8 v3.0 expression beadchip</td><td colspan="4">
<table>
<tr><th>Gene symbol</th><th>ProbeID</th><th>Average control cells[III]</th><th>Average Ago2 siRNA treated cells[III]</th><th>Average UPF1 and Ago2 siRNA treated cells[III]</th><th>T-Test control – Ago2</th><th>T-Test control - UPF1+Ago2</th></tr>
<tr><td>HMGB1</td><td>ILMN_2231242</td><td>7.17</td><td>7.09</td><td>7.08</td><td>0.59</td><td>0.69</td></tr>
<tr><td>UTP14C</td><td>ILMN_1686645</td><td>8.94</td><td>8.78</td><td>8.63</td><td>0.30</td><td>0.05</td></tr>
<tr><td>FRRS1</td><td>ILMN_2214734</td><td>7.00</td><td>6.88</td><td>6.93</td><td>0.42</td><td>0.64</td></tr>
<tr><td>LOC401052</td><td>ILMN_1791423</td><td>7.89</td><td>8.21</td><td>8.00</td><td>0.16</td><td>0.81</td></tr>
<tr><td>MGC119295</td><td>ILMN_2144654</td><td>6.96</td><td>7.03</td><td>6.94</td><td>0.37</td><td>0.91</td></tr>
<tr><td>LOC442578</td><td>ILMN_1791375</td><td>11.57</td><td>11.45</td><td>11.63</td><td>0.47</td><td>0.68</td></tr>
</table>
</td></tr>
<tr><td>GSE20491</td><td>Duns, G. et al, Cancer Res 70(11):4287-4291 (2010) [V].
PMID:20501857

10 clear cell renal cell</td><td>HMGB1</td><td>ILMN_2231242 and ILMN_1791466 probes both identify NM_002128.4</td><td>
<table>
<tr><th>Cell line</th><th>Control Average</th><th>Caffeine Average</th><th>Emetine Average</th></tr>
<tr><td>RCC1</td><td>8.6</td><td>8.1</td><td>7.9</td></tr>
<tr><td>RCC4</td><td>7.8</td><td>7.5</td><td>7.5</td></tr>
<tr><td>RCC5</td><td>6.8</td><td>6.8</td><td>6.7</td></tr>
<tr><td>RCC-AB</td><td>8.9</td><td>-</td><td>8.2</td></tr>
</table>
</td><td>
<table>
<tr><th>control-caffeine</th><th>control-emetine</th></tr>
<tr><td>0.06</td><td>0.04</td></tr>
<tr><td>0.27</td><td>0.37</td></tr>
<tr><td>0.99</td><td>0.07</td></tr>
<tr><td>-</td><td>0.01</td></tr>
</table>
</td></tr>
</table>

	carcinoma cell lines; emetine or caffeine treated.<br><br>Illumina HumanHT-12 V3.0 expression beadchip			RCC-ER	7.2	6.7	6.9	0.07	0.3
				RCC-FG2	8.0	7.7	7.4	0.49	0.18
				RCC-HS	8.7	8.0	8.1	0.0009	0.013
				RCC-JF	8.0	7.5	7.6	0.15	0.25
				RCC-JW	8.1	7.4	7.7	0.16	0.46
				RCC-MF	7.6	6.9	6.9	0.27	0.22
		UTP14C	ILMN_1686645 identifies transcript NM_021645	Cell line	Control Average	Caffeine Average	Emetine Average	control-caffeine	control-emetine
				RCC1	7.8	7.7	7.5	0.46	0.02
				RCC4	8.2	7.9	8.2	0.18	0.99
				RCC5	7.7	7.9	7.9	0.04	0.29
				RCC-AB	8.7	-	8.7	-	0.9
				RCC-ER	8.5	8.1	8.0	0.06	0.01
				RCC-FG2	8.0	7.9	7.8	0.52	0.31
				RCC-HS	7.8	7.7	7.7	0.61	0.56
				RCC-JF	8.8	8.9	8.4	0.09	0.02
				RCC-JW	8.0	8.0	8.0	0.08	0.22
				RCC-MF	8.4	8.0	7.4	0.33	0.04

GSE24204	Mattila, H., University of Tampere. Finland (unpublished)<br><br>peripheral mononuclear leukocytes cell lines from healthy and prostate cancer patients; Emetine treated cells; normalized log10 ratio emetine treated/control cells	C20orf203	27463 - Identifies transcript AK091025	Healthy average expression ratio: -0.15<br>Cancer average expression ration: -0.18	In this study, a considered gene was declared differentially expressed if the calculated log10 ratio value was greater than zero. Black labeled results display values smaller than zero indicating on a
		HMGB1	27795, 2170, 7063 and 8395 probes - identify transcript NM_002128	Healthy average expression ratio: -0.07<br>Cancer average expression ration: -0.07	
		UTP14C	32662 - identifies transcript NM_021645;	Healthy average expression ratio: 0.49<br>Cancer average expression ration: 0.48	
		ZNF841	39976 - identifies transcript NM_001136499	Healthy average expression ratio: 0.41<br>Cancer average expression ration: 0.45	
		TXNDC6	7699, 11753 and 4719 probes – identify transcript NM_178130	Healthy average expression ratio: -0.03<br>Cancer average expression ration: -0.01	
		FRRS1	31823 - identifies transcript NM_001013660	Healthy average expression ratio: -0.03<br>Cancer average expression ration: 0.01	

	Agilent-012391 Whole Human Genome Oligo Microarray G4112A	LOC401052	13485 - identifies transcript NM_001008737	Healthy average expression ratio: -0.03<br>Cancer average expression ration: -0.02	down-regulation effect after exposure to emetine.
		ERVFRD-1	14886 - identifies transcript NM_207582	Healthy average expression ratio: -0.15<br>Cancer average expression ration: -0.25	
		STAG3L3	3563 - identifies transcript NM_001013739	Healthy average expression ratio: -0.10<br>Cancer average expression ration: -0.11	
GSE29788	Sharma. S., et al, Mol Cancer Ther. 10(9):1751-1759,(2011)<br>PMID: 21764905<br><br>Primary human head and neck cancer cell line (SCC12) and matched normal controls; Emetine treated cells; probes which are marked as absent in all cells were not considered.<br><br>Affymetrix Human Genome U133A Array	HMGB1	200679_x_at and 200680_x_at identify transcript NM_002128 (3428 bp);<br>214938_x_at identifies also AF283771 (1814 bp, partially similar to HMGB1, encoded on the opposite strand of the DNA)	200679_x_at=1.02<br>200680_x_at=1.12<br>214938_x_at=1.96	NEI <1.7 (nonsense enrichment Index) indicate on NMD- immune transcript.
		UTP14C	203614_at – identifies NM_021645 transcript;<br>221513_s_at - identifies both UTP14C (chr13) and UTP14 (chrX) genes.	203614_at=1.36<br>221513_s_at=1.27	
		ZNF117	207117_at and 207605_x_at<br>- identify NM_015852 transcript;	207117_at=0.86<br>207605_x_at=1.0	

[I] If for a given gene the array included several probes, probes which identify monocistronic transcripts solely are not indicated (no NMD effect was observed in the listed experiments for those probes).

[II] NM_005678 – bicistronic, the longer variant includes exons 1-10, is the predominant form of this gene; additional monocistronic and bicistronic variants are known.

[III] Normalized intensity values are shown on a log 2 scale.

[IV] the MTPN CDS ends at the terminal exon therefore this transcript is NMD-immune by definition. LUZP6 CDS is positioned within the 3' UTR of the transcript, at the terminal exon.

[V] levels below a value of 7 are close to detection limit; hence bicistronic gene probes indicating expression bellow 7 are not listed.

[VI] down-regulation differential expression is detected, thus does not indicate on NMD sensitivity of the transcript.
